# Research on the correlation and potential mechanism of PKCδ expression with efficacy and prognosis in diffuse large B-cell lymphoma

**DOI:** 10.3389/fonc.2026.1690426

**Published:** 2026-02-06

**Authors:** Xulu Zhao, Shan Li, Lin Zhu, Mei Wu, Xin Hu, Xiao Liang, Shanshan Wang, Aziguli Maihemaiti, Abulikemujiang Adili, Shujuan Wen

**Affiliations:** Department of Lymphoma, Cancer Hospital Affiliated to Xinjiang Medical University, Urumqi, China

**Keywords:** cancer treatment, diffuse large B-cell lymphoma, efficacy, PKC-δ, prognosis, rottlerin, therapeutic resistance

## Abstract

**Introduction:**

Diffuse large B-cell lymphoma (DLBCL) is the most common subtype of non-Hodgkin lymphoma. While the R-CHOP regimen achieves a 70% 5-year survival rate, patients with refractory or relapsed disease face poor prognoses. Therefore, it is very important to search for markers related to curative effect and prognosis, and to explore new targeted therapies. Protein kinase C delta (PKCδ), a serine/threonine kinase involved in cell proliferation, growth, and cancer progression, has been proposed as a prognostic marker in solid tumors, but its role in DLBCL remains underexplored. This study aimed to validate PKCδ as a prognostic biomarker and investigate its mechanistic contributions to therapeutic resistance.

**Methods:**

Immunohistochemistry (IHC) was used to analyze the expression of PKCδ in 200 DLBCL tissues to validate the correlation between PKCδ and therapeutic efficacy as well as prognosis. Using the DB and RIVA cell lines to stably knock down the PRKCD gene, we explored the role of PKCδ in cell proliferation, cell cycle, apoptosis, chemoresistance, and related signaling pathways through CCK-8 assays, flow cytometry, RNA sequencing, and in vivo xenograft models in nude mice. Additionally, we evaluated the therapeutic efficacy of the multi-kinase inhibitor Rottlerin both in vitro and in vivo.

**Results:**

High PKCδ expression correlated with reduced 5-year progression-free survival and overall survival. Knockout of PKCδ repressed DLBCL cell proliferation, facilitated cell cycle arrest in the G2/M phase, induced apoptosis in vitro, and inhibited tumor growth in vivo, and enhanced sensitivity to rituximab and chemotherapeutics. Similarly, inhibition with the multi-kinase inhibitor Rottlerin also impaired tumor growth and showed combinatory efficacy with rituximab. RNA-seq revealed 2,988 differentially expressed genes enriched in AKT, MAPK, and NF-κB signaling pathways.

**Discussion:**

Our findings highlight PKCδ as a potential predictive biomarker and therapeutic target. However, due to the off-target effects of Rottlerin, the observed in vivo efficacy and synergistic effects of Rottlerin should be considered as preliminary pharmacological support for the concept of targeting PKCδ.

## 1 Introduction

Diffuse large B-cell lymphoma (DLBCL) is the most common subtype of lymphoma, accounting for approximately 30–40% of non-Hodgkin lymphoma cases (1). Despite the significant efficacy of R-CHOP chemotherapy (rituximab, cyclophosphamide, doxorubicin, vincristine, and prednisone), 30–40% of patients still experience refractory or relapsed disease with poor prognosis (2–4), underscoring the urgent need for novel targeted therapies with innovative mechanisms of action, new drug combinations, predictive and novel therapeutic strategies (5).

The protein kinase C (PKC) family, as a serine/threonine kinase, is widely present in various tissues and has diverse biological functions. Protein kinase C delta (PKCδ), a member of the PKC family. The critical role of PKCδ dysregulation in tumor promotion has been extensively described elsewhere (6, 7), regulates critical cellular processes, including proliferation, apoptosis, and drug resistance during cancer progression (8–10). PKCδ may target the reprogramming of mononuclear phagocytes to enhance immunotherapy in various cancers. PKCδ plays a key role in controlling mononuclear phagocyte-induced effector T cell suppression and subsequent tumor promotion. Genetic deletion of PKCδ can inhibit tumor growth and promote T cell tumor infiltration and activation (11). PKCδ inhibition can affect the proliferation of breast cancer cells but has no impact on breast cancer stem cells. Moreover, activation of the Ras/MAPK signaling pathway can help breast cancer stem cells evade the effects of certain stem cell-targeted therapies (12). Research indicates that PKCδ facilitates acquired resistance to EGFR inhibitors through stabilizing the interaction between sodium/glucose cotransporter 1 (SGLT1) and EGFR, as well as enhancing glucose uptake (13). High expression of PKCδ in pancreatic cancer is associated with low survival rates (14). A comprehensive review by Kawano et al. has highlighted the complex, context-dependent role of PKCδ in lymphoma, particularly its ability to trigger apoptosis in response to chemotherapeutic agents (15). However, comprehensive studies validating its clinical prognostic value and exploring its role in therapeutic resistance, especially to monoclonal antibodies like rituximab in DLBCL, remain limited.

The prospect of comprehending and developing PKCδ-targeted cancer therapies holds extensive potential. In this study, we investigated the prognostic value of PKCδ in DLBCL and its mechanistic contributions to therapeutic resistance, the ability of PKCδ inhibition to trigger apoptosis in DLBCL cells, the activation of survival signaling pathways mediated by PKCδ, and the impact of the PKCδ inhibitor rottlerin on drug or chemotherapy sensitivity. We hypothesized that PKCδ inhibition sensitizes DLBCL cells to rituximab and chemotherapy by modulating critical signaling pathways and assessed the therapeutic potential of PKCδ inhibition both *in vitro* and *in vivo*.

## 2 Materials and methods

### 2.1 Patient data and ethical approval

We collected tissue blocks and clinical data from 200 patients who were initially treated and pathologically diagnosed with DLBCL at the Affiliated Cancer Hospital of Xinjiang Medical University between 2019 and 2022. The pathological diagnostic criteria were based on the 2008 World Health Organization classification of lymphomas (16). Clinical staging was performed using the Ann Arbor staging system (17). Patient risk stratification was based on the International Prognostic Index (IPI) (18), with patients classified into four groups: low-risk group (0–1 points), low-intermediate-risk group (2 points), intermediate-high-risk group (3 points), and high-risk group (4–5 points). The DLBCL cases were classified into two subtypes using the Hans classification method: Germinal center B-like (GCB) and non-GCB. Patient performance status was assessed using the Eastern Cooperative Oncology Group (ECOG) score. Extranodal involvement is defined as the presence of lymphoma in organs or tissues outside the lymph nodes. The first-line chemotherapy regimens were CHOP/CHOP-like, consisting of cyclophosphamide, anthracycline or liposomal doxorubicin, vincristine, and prednisolone, with a 21-day treatment cycle; or R-CHOP/R-CHOP-like, consisting of rituximab, cyclophosphamide, anthracycline or liposomal doxorubicin, vincristine, and prednisolone, with a 21-day treatment cycle. A subset of patients in our study cohort received only CHOP chemotherapy due to economic barriers that limited access to the drug. Progression-free survival (PFS) is defined as the time from the start of treatment to disease progression or death (from any cause). Overall survival (OS) is defined as the time from the start of treatment to death from any cause or the last follow-up. All DLBCL samples were obtained from Xinjiang Medical University Affiliated Tumor Hospital. This research was endorsed by the Institutional Review Board and the ethics committees of Xinjiang Medical University Affiliated Tumor Hospital. Written informed consents were acquired from all contributors prior to treatment to gain tissue samples and medical information. The research was conducted following the directions of the Declaration of Helsinki.

### 2.2 Patient samples and immunohistochemistry

Formalin-fixed paraffin-embedded (FFPE) tissues from 200 DLBCL patients diagnosed with diffuse large B-cell lymphoma (DLBCL) at Xinjiang Medical University Affiliated Tumor Hospital (2019–2022) were sectioned (4 μm). Immunohistochemical (IHC) staining was performed using anti-PKCδ antibody (Abcam, Cat# ab182126, 1:200 dilution) to detect the expression levels of PKCδ protein in the tissues. Under an optical microscope, the staining intensity and percentage of positive cells were observed to determine the scores. The percentage of positive cells was scored as follows: 1 = 0-4%, 2 = 5-19%, 3 = 20-39%, 4 = 40-59%, 5 = 60-79%, 6 = 80-100%. The staining intensity was scored as follows: 0=no staining, 1=week staining, 2=intermediate staining, 3=strong staining. The product of the scores for staining intensity and percentage of positive cells was considered the final score. Cases with a final score of ≥4 were classified as high expression, while others were classified as low expression.

### 2.3 Cell lines

The germinal center B-cell-like (GCB) DLBCL cell lines SUDHL-4 and DB, and the non-germinal center B-cell-like (non-GCB) DLBCL cell lines RIVA and U2932, and IM-9 (human peripheral blood B lymphocytes, lymphoblastoid) were purchased from Yaji Biotechnology, which were cultured in RPMI-1640 (Procell, Wuhan) medium supplemented with 10% fetal bovine serum (Excell Bio, China, FND500) and 1% PBS (ZSGB-BIO, Beijing), the incubation conditions were 37°C with 5% carbon dioxide (CO2).

### 2.4 Establishment of stable knockout of PKCδ cell lines

To knock down the expression of PKCδ protein, we established stable cell lines using shRNA targeting the PRKCD gene. The sequence TTCTCCGAACGTGTCACGT was employed as the RNAi negative control (Negative Control, NC) scramble sequence. The target sequences were engineered into the respective lentiviral vectors. Three target sequences were designed based on the CDS region sequence of the PRKCD gene for base synthesis. The RNAi target sequences are delineated in **Table 1**. 293T cells were transfected with plasmids (GV112 and GV493, GeneChem) to package lentiviruses, and the viral titer was tested. DB and RIVA cells were transfected with lentivirus at a multiplicity of infection (MOI) of 100, and stable clones were selected with 1.5 μg/mL puromycin (Sigma-Aldrich).

**Table 1:** Description of homo sapiens protein kinase C delta (PKCδ), transcript variant 2, mRNA.

NO.	Accession	Target seq	CDS	GC%
PKCδ-RNAi(72998)	NM_212539	ggCCGCTTTGAACTCTACCGT	240.2270	52.63%
PKCδ-RNAi(72999)	NM_212539	gcAGGGATTAAAGTGTGAAGA	240.2270	36.84%
PKCδ-RNAi(73000)	NM_212539	caAGGCTACAAATGCAGGCAA	240.2270	47.37%

### 2.5 Western Blot

Cells were collected at a concentration of 1×10^6 cells, washed with PBS, and lysed in 100 μL RIPA buffer (supplemented with protease and phosphatase inhibitors) by homogenization on ice for 60 minutes, followed by centrifugation at 12,000 rpm for 4°C. Protein concentration was determined using the BCA method (Easy II Protein Quantitative Kit, TransGen Biotech, DQ111-01). Proteins were separated by SDS-PAGE and transferred to PVDF membranes (Millipore, IPVH00010). The membranes were blocked and then incubated with primary antibodies against PKCδ (1:800) Rabbit mAb (#9616, CST), β-Actin (Mouse mAb, Sino Biological, 1:1000) Phospho-NF-kB p65 (Ser536) (Rabbit mAb, Affinity, AF2006), Phospho-AKT (Ser473) (Rabbit mAb, proteintech, 66444-1-Ig), Phospho-Erk1/2 (Thr202/Tyr204) (Rabbit mAb, proteintech, 11257-1-AP), and IKB alpha (Rabbit mAb, Affinity, AF7776) overnight at 4 °C. After washing, the membranes were incubated with corresponding goat anti-rabbit IgG H&L (HRP) (ab205718, Abcam, 1:5000) and goat anti-mouse IgG H&L (HRP) (ab205719, Abcam, 1:15000). Detection was performed using the SuperSignal™ West Pico PLUS Chemiluminescent Substrate (Thermo Fisher, 34580) and visualized with the ChemiScope 300 chemiluminescence imager.

### 2.6 Quantitative real-time polymerase chain reaction

Total RNA, utilized for RT-qPCR assay, was extracted from cells using the TRIzol reagent (Thermo Fisher Scientific) following the manufacturer’ protocol. PrimeScript™ RT Reagent Kit (TaKaRa) was employed for the synthesis of complementary DNA(cDNA) from total RNA. Subsequently, RT-qPCR was conducted using the SYBR^®^ Premix Ex Taq™ II Kit (TaKaRa), adhering to the manufacturer’s protocol. The expression of the target gene (PRKCD) was normalized to the endogenous reference gene β-actin. The calculation of relative gene expression was performed using the 2-ΔΔCT method.

### 2.7 CCK-8

Selected wells were augmented with CCK-8 reagent (APExBIO, Houston, USA). Cells were treated with rituximab (10–100 μg/mL), doxorubicin (0.1–1.6 μg/mL), or cisplatin (0.2–3.2 μg/mL) at 37 °C for 24, 48, or 72 hours, a microplate reader (Bio-Rad, China) was utilized to assess absorbance values at 450 nm.

### 2.8 RNA sequencing and pathway analysis

Total RNA was extracted from four stable PRKCD-knockout DB and RIVA cells and their non-target controls using RNAiso Plus (TaKaRa) for the construction of sequencing library. RNA-seq library were sequenced on an Illumina NovaSeq 6000 (150 bp paired-end). Differentially expressed genes (|log2 fold change|>1, p<0.05) were identified using “DESeq2” R package and analyzed via KEGG/GO enrichment (ClusterProfiler).

### 2.9 Drug sensitivity assays

Drug sensitivity was assessed using CCK-8 assays in PRKCD-knockout DB (germinal center B-cell-like DLBCL) and RIVA (non-germinal center B-cell-like DLBCL) cell lines. The IC50 values for doxorubicin, cisplatin, and rituximab were determined through dose-response curves, and non-toxic IC25 doses were selected for subsequent experiments. DB and RIVA cells were treated with doxorubicin, cisplatin, or rituximab at non-toxic IC25 doses for 24h, 48h, and 72h to evaluate the time-dependent effects of PRKCD-knockout on drug sensitivity. Cell proliferation rates were measured in three groups: blank control, shRNA-NC (negative control), and PRKCD-shRNA.

### 2.10 *In Vivo* xenograft models

A total of 80 female BALB/c nude mice of SPF grade (Animal Quality Certificate Number: 20221128Abzz0100018442), aged 5–6 weeks and weighing 20–22 g, were purchased from Hangzhou Medical College (Experimental Animal Production License Number: SCXK (Zhejiang) 2019-0002; Experimental Animal Use License Number: SYXK (Zhejiang) 2019-0011). The nude mice were maintained at a room temperature of 22 ± 2°C, with relative humidity of 60%-80%, under a diurnal cycle, and were allowed free access to food and water. The experimental groups are shown in **Table 2**. Starting from the day of cell injection, the body weight of the nude mice was measured every 5 days. The maximum length (a) and width (b) of the subcutaneous tumor were measured using a caliper. The tumor volume was calculated using the formula (volume = 1/2*ab^2) to analyze the changes in tumor volume during the experiment. After the experiment was completed, nude mice were euthanized by cervical dislocation performed by appropriately trained researchers without anesthesia. The tumor tissues were surgically excised, weighed, and photographed. The tumor tissues were divided into two portions, with one portion being fixed in 10% neutral formalin and the other being rapidly frozen at -80°C. The tumor growth inhibition rate (IR) was calculated using the formula: IR = (average tumor weight of the control group - experimental group)/average tumor weight of the control group×100%. The animal experimental procedures followed the institutional ethical guidelines and were approved by Xinjiang Medical University Affiliated Tumor Hospital (Approval no:81860042).

**Table 2:** Drug administration methods and doses for each experimental group.

Different groups	Cells	Method	Vehicle (dose)
Blank group	DB	Gavage	Equal volume of solvent (0.5% CMC-Na)
Rottlerin treatment group	DB	Gavage	PRKCD inhibitor (20 mg/kg)
Rituximab treatment group	DB	Intraperitoneal injection	Rituximab (20 mg/kg/day)
Rottlerin + Rituximab group	DB	Gavage	PRKCD inhibitor (20 mg/kg)
Negative control shRNA group	shRNA negative control stable expression strain	Gavage	Equal volume of solvent (0.5% CMC-Na)
NC shRNA + Rituximab group	shRNA negative control stable expression strain	Intraperitoneal injection	Rituximab (20 mg/kg/day)
PRKCD-shRNA group	PRKCD-shRNA stable expression strain	Gavage	Equal volume of solvent (0.5% CMC-Na)
PRKCD-shRNA + Rituximab group	PRKCD-shRNA stable expression strain	Intraperitoneal injection	Rituximab (20 mg/kg/day)

### 2.11 Hematoxylin and eosin staining and terminal deoxynucleotidyl transferase dUTP nick end labeling staining

After the tumor tissues were excised from the nude mice, they were first rinsed with normal saline to remove residual blood and then fixed in 10% neutral formalin solution. The fixed tissues were subsequently embedded in paraffin, sectioned, and stained with hematoxylin and eosin (HE). In addition, TUNEL staining was performed to detect cellular apoptosis. According to the instructions of the TUNEL apoptosis detection kit (Boster), a labeling working solution was prepared by mixing 1 μl of TdT enzyme with 1 μl of DIG-d-UTP in 18 μl of labeling buffer. The sections were subjected to blocking, incubation with diluted antibodies, staining, and finally mounted with neutral gum for microscopic observation and analysis.

### 2.12 Flow cytometry analysis

Cell cycle and apoptosis were detected by flow cytometry (Navios, Beckman Coulter, CA, USA). For cell cycle analysis, after collection and washing twice with PBS, the DLBCL cells were fixed with 70% ethanol at -20°C overnight, followed by resuspending in PI/RNase staining solution (BD Biosciences, MA, USA). After incubating the samples(37°C, 30min), the labeled cells were analyzed via flow cytometry (BD FACS Canto II), cell apoptosis was analyzed using Annexin V-PE/7AAD assay staining (BD Biosciences), following the manufacturer’s instructions (19). The data was processed with Modfit5 to calculate the proportion of cells in distinct cell cycle phases.

### 2.13 Statistical analysis

Statistical analysis was performed using SPSS 19.0. Quantitative data were tested for normality using the Shapiro-Wilk (SW) test. Data with a normal distribution were shown as mean ± standard deviation (X ± SD) and analyzed by one-way ANOVA. For follow-up pairwise comparisons, the Turkey test was used if variances were equal, and Dunnett’s-T3 test was used if variances were unequal. Data without a normal distribution were presented as median (M) with the interquartile range (P25–P75) and analyzed by the Kruskal-Wallis H test. For further pairwise comparisons, the P values were adjusted using the Bonferroni method. P value below 0.05 was taken as statistically significant. Survival curves were generated using Kaplan-Meier analysis. Data are presented as mean ± SD. Differences were assessed via Student’s t-test or ANOVA(p<0.05). GraphPad Prism 5.0 was used to create the figures.

## 3 Results

### 3.1 PKCδ was upregulated in DLBCL and correlated with poor prognosis

**Figure 1:**
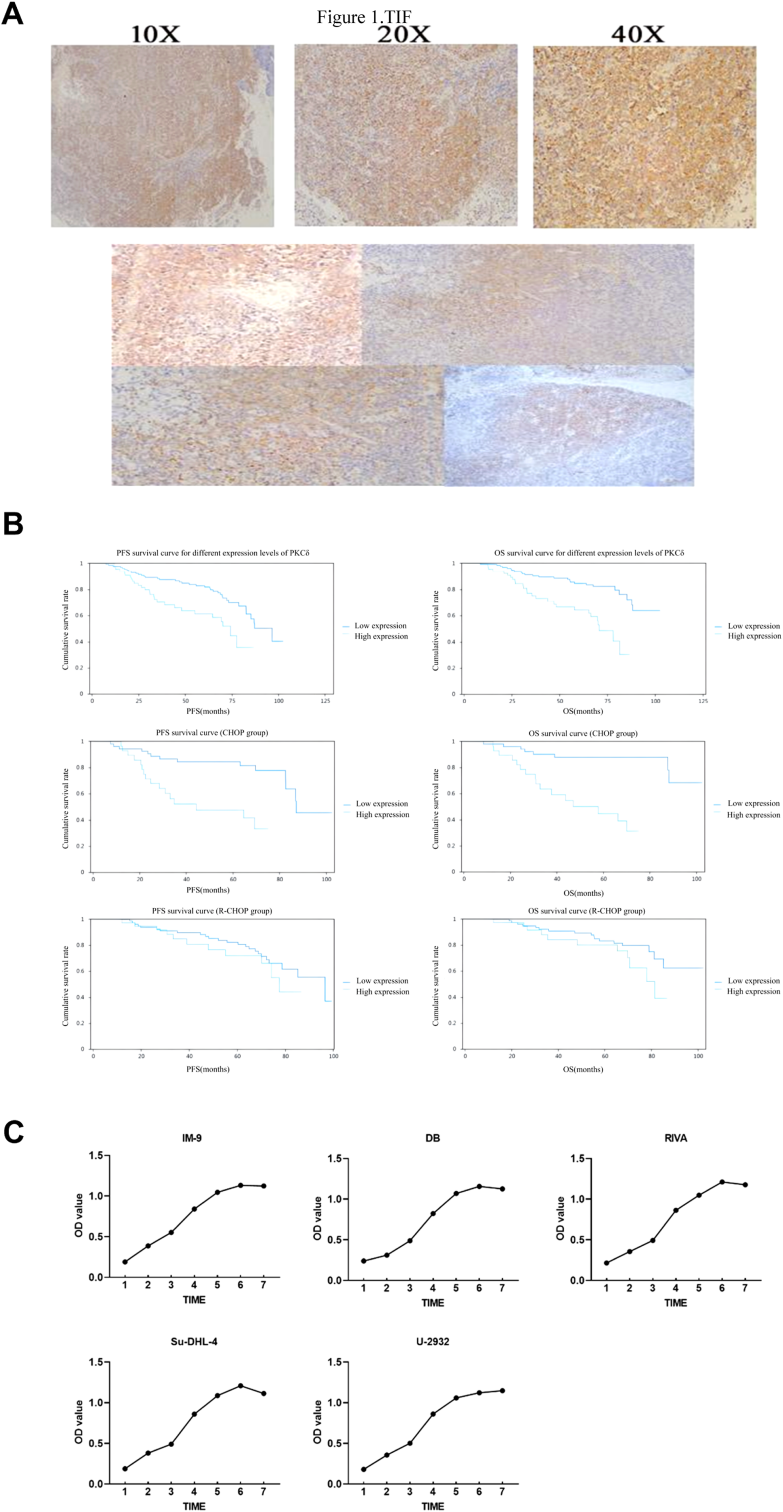
**(A)** Representative IHC images showing PKCδ expression in the tissues. **(B)** Kaplan-Meier survival curves for high and low expression of PKCδ among 200 DLBCL patients, the group receiving CHOP chemotherapy and rituximab monoclonal antibody combined with CHOP regimen. CHOP,cyclophosphamide, doxorubicin, vincristine, and prednisone; R-CHOP, rituximab, cyclophosphamide, doxorubicin, vincristine, and prednisone; PFS, progression­free survival;OS, overall survival. **(C)** Growth curve of IM-9 cells and lymphoma cell lines DB, RIVA, Su-DHL-4, and U-2932 at different time points.

In this study, 33.5% (67/200) of the diffuse large B-cell lymphoma patient samples showed high expression of PKCδ protein, with positive membrane staining demonstrated by immunohistochemistry (**Figure 1A**). The 200 DLBCL patients were divided into two groups: high expression of PKCδ and low expression of PKCδ. The correlation between PKCδ expression and the therapeutic effect and prognosis of DLBCL was analyzed. Comparison of baseline characteristics showed that patients in the high PKC-δ expression group had a significantly worse ECOG performance status than those in the low expression group (86.57% vs 72.93%, P = 0.029). There were no statistically significant differences between the two groups in terms of gender, age, Ann Arbor stage, IPI score, extranodal involvement, treatment cycles, LDH levels, whether they received rituximab treatment, and cell of origin (P>0.05) (**Table 3**). The CHOP and R-CHOP groups are well-balanced in most baseline characteristics, including sex, age, stage, PKCδ expression, LDH level, treatment cycles and histological subtype. However, there are statistically significant differences in IPI score, extranodal involvement, and ECOG score (**Supplementary Table S1**). Univariate analysis results showed that high PKCδ expression, clinical stage III-IV, and treatment cycles of 4–7 weeks, ≥8 weeks were factors affecting the OS and PFS of patients (P<0.05). Multivariate Cox analysis results showed that treatment cycles of 4–7 weeks, ≥8 weeks were protective factors for OS and PFS in patients. High PKCδ expression was an independent risk factor affecting the OS and PFS of patients (P<0.05) (**Tables 4**, **5**). The subgroup analysis results showed that for overall survival (P for interaction = 0.020), the interaction test for whether patients received rituximab treatment was statistically significant. Among patients who received only CHOP chemotherapy, high PKCδ expression was associated with significantly worse overall survival (HR = 7.50, 95% CI: 2.60-21.67, P < 0.001). In contrast, among patients treated with the R-CHOP regimen, the impact of high PKCδ expression on overall survival was attenuated and not statistically significant (HR = 1.46, 95% CI: 0.60-3.51, P = 0.403) (**Table 6**). A consistent trend was observed in the analysis of progression-free survival (P for interaction = 0.035) (**Table 7**). This result indicates that rituximab monoclonal antibody treatment may reverse the adverse prognostic factor of high PKCδ expression.

**Table 3:** Comparison of baseline characteristics between low and high PKCδ expression groups(n, %).

Characteristics	Total (n = 200)	PKCδ negative (n = 133)	PKCδ positive (n = 67)	χ²	P
sex				0.30	0.584
female	92 (46.00)	63 (47.37)	29 (43.28)		
male	108 (54.00)	70 (52.63)	38 (56.72)		
Age(years)				1.36	0.243
≤60	148 (74.00)	95 (71.43)	53 (79.10)		
>60	52 (26.00)	38 (28.57)	14 (20.90)		
Ann Arbor stage				1.82	0.178
I-II	94 (47.00)	67 (50.38)	27 (40.30)		
III-IV	106 (53.00)	66 (49.62)	40 (59.70)		
IPI				3.90	0.273
0-1分	99 (49.50)	63 (47.37)	36 (53.73)		
2分	53 (26.50)	33 (24.81)	20 (29.85)		
3分	39 (19.50)	29 (21.80)	10 (14.93)		
4-5分	9 (4.50)	8 (6.02)	1 (1.49)		
Extranodal involvement				0.38	0.538
0-1	153 (76.50)	100 (75.19)	53 (79.10)		
≥2	47 (23.50)	33 (24.81)	14 (20.90)		
Cycles(week)				3.50	0.174
<4	37 (18.50)	29 (21.80)	8 (11.94)		
4~7	136 (68.00)	85 (63.91)	51 (76.12)		
≥8	27 (13.50)	19 (14.29)	8 (11.94)		
ECOG scores				4.75	0.029
0-1	45 (22.50)	36 (27.07)	9 (13.43)		
≥2	155 (77.50)	97 (72.93)	58 (86.57)		
LDH level				0.03	0.868
Normal	124 (62.00)	83 (62.41)	41 (61.19)		
Abnormal	76 (38.00)	50 (37.59)	26 (38.81)		
Rituximab containing				0.07	0.792
no	81 (40.50)	53 (39.85)	28 (41.79)		
yes	119 (59.50)	80 (60.15)	39 (58.21)		
Histologic category				0.20	0.654
GCB	97 (48.50)	66 (49.62)	31 (46.27)		
non-GCB	103 (51.50)	67 (50.38)	36 (53.73)		

**Table 4:** Univariate and multivariate analyses of factors influencing the OS of patients.

Factors	Univariate analysis			Multivariate analysis		
	β	HR (95%CI)	*P*	β	HR (95%CI)	*P*
Sex, male	-0.44	0.65 (0.37 ~ 1.12)	0.118			
Age, >60 years	-0.51	0.60 (0.30 ~ 1.20)	0.150			
Stage, III-IV	0.66	1.93 (1.09 ~ 3.41)	0.025	0.52	1.68 (0.94 ~ 2.99)	0.079
IPI						
0-1		1.00 (Reference)				
2	-0.07	0.93 (0.49 ~ 1.78)	0.829			
3	-0.02	0.98 (0.47 ~ 2.04)	0.967			
4-5	-0.05	0.95 (0.22 ~ 4.01)	0.941			
Extranodal involvement ≥2	-0.04	0.96 (0.51 ~ 1.81)	0.907			
Cycles						
<4		1.00 (Reference)			1.00 (Reference)	
4~7	-0.81	0.45 (0.22 ~ 0.89)	0.022	-0.84	0.43 (0.21 ~ 0.86)	0.018
≥8	-1.17	0.31 (0.12 ~ 0.81)	0.017	-1.05	0.35 (0.13 ~ 0.92)	0.034
ECOG ≥2	0.34	1.40 (0.68 ~ 2.88)	0.358			
LDH, elevated	0.15	1.16 (0.66 ~ 2.03)	0.604			
PKCδ positive	1.18	3.27 (1.86 ~ 5.75)	<0.001	1.16	3.20 (1.80 ~ 5.66)	<0.001
Rituximab containing	-0.32	0.72 (0.42 ~ 1.25)	0.246			
non-GCB subtype	-0.08	0.93 (0.54 ~ 1.60)	0.781			

**Table 5:** Univariate and multivariate analyses of factors influencing the PFS of patients.

Factors	Univariate analysis			Multivariate analysis		
	β	HR (95%CI)	*P*	β	HR (95%CI)	*P*
Sex, male	-0.43	0.65 (0.40 ~ 1.06)	0.086			
Age, >60 years	-0.44	0.65 (0.35 ~ 1.19)	0.159			
Stage, III-IV	0.63	1.89 (1.13 ~ 3.14)	0.015	0.51	1.67 (1.00 ~ 2.80)	0.051
IPI						
0-1		Reference				
2	0.05	1.05 (0.59 ~ 1.86)	0.871			
3	0.04	1.04 (0.55 ~ 2.00)	0.896			
4-5	-0.23	0.80 (0.19 ~ 3.35)	0.758			
Extranodal involvement ≥2	0.01	1.00 (0.58 ~ 1.75)	0.992			
Cycles						
<4		Reference			1.00 (Reference)	
4~7	-0.89	0.41 (0.22 ~ 0.77)	0.006	-0.90	0.41 (0.21 ~ 0.78)	0.006
≥8	-0.90	0.41 (0.18 ~ 0.90)	0.027	-0.82	0.44 (0.20 ~ 0.98)	0.045
ECOG ≥2	0.28	1.32 (0.71 ~ 2.48)	0.381			
LDH, elevated	0.08	1.08 (0.65 ~ 1.80)	0.754			
PKCδ positive	0.81	2.25 (1.35 ~ 3.74)	0.002	0.82	2.26 (1.35 ~ 3.79)	0.002
Rituximab containing	-0.34	0.71 (0.44 ~ 1.16)	0.172			
non-GCB subtype	-0.16	0.85 (0.52 ~ 1.39)	0.519			

**Table 6:** Subgroup analysis of the impact of high PKCδ expression on OS.

Factors	n	PKCδ negative	PKCδ positive	HR (95%CI)	*P*	P for interaction
All patients	200	25/133	27/67	3.27 (1.86 ~ 5.75)	<0.001	
Age(years)						0.145
≤60	148	21/95	21/53	2.43 (1.31 ~ 4.51)	0.005	
>60	52	4/38	6/14	9.28 (2.26 ~ 38.05)	0.002	
Rituximab containing						0.02
no	81	8/53	16/28	7.50 (2.60 ~ 21.67)	<0.001	
yes	119	17/80	11/39	1.46 (0.60 ~ 3.51)	0.403	
Histologic category						0.681
GCB	97	12/66	14/31	3.76 (1.69 ~ 8.34)	0.001	
non-GCB	103	13/67	13/36	2.82 (1.26 ~ 6.32)	0.012	

HR, the hazard ratio of the high PKCδ expression group compared to the low expression group. P value for interaction was calculated using a Cox regression model with an interaction term.

**Table 7:** Subgroup analysis of the impact of high PKCδ expression on PFS.

Factors	n	PKCδ negative	PKCδ positive	HR (95%CI)	*P*	P for interaction
All patients	200	38/133	27/67	2.25 (1.35 ~ 3.74)	0.002	
Age(years)						0.146
≤60	148	31/95	21/53	1.75 (0.98 ~ 3.10)	0.056	
>60	52	7/38	6/14	6.10 (1.89 ~ 19.63)	0.002	
Rituximab containing						0.035
no	81	14/53	16/28	4.03 (1.81 ~ 8.95)	<0.001	
yes	119	24/80	11/39	1.39 (0.67 ~ 2.87)	0.374	
Histologic category						0.958
GCB	97	20/66	14/31	2.36 (1.17 ~ 4.79)	0.017	
non-GCB	103	18/67	13/36	2.15 (1.02 ~ 4.54)	0.045	

HR, the hazard ratio of the high PKCδ expression group compared to the low expression group. P value for interaction was calculated using a Cox regression model with an interaction term.

In patients with high and low expression of PKCδ in DLBCL, the 3-year PFS was 70.5% and 87.6% (p<0.01), respectively; the 3-year OS was 75.2% and 90.5% (p<0.01), respectively; the 5-year PFS was 61.4% and 81.9% (p<0.01), respectively; and the 5-year OS was 64.4% and 84.7% (p<0.01), respectively (**Figure 1B**). In patients with high and low expression of PKCδ in DLBCL, the objective response rate (ORR) was 83.5% and 93.2% (p<0.05), respectively (**Table 8**). These results suggest that patients with high expression of PKCδ have poorer prognosis and therapeutic outcomes. Among patients who only received CHOP regimen chemotherapy, those with high expression of PKCδ had a significantly worse prognosis than those with negative PKCδ expression. The 5-year PFS was 47.5% and 84.4% respectively(p<0.01), and the 5-year OS was also 44.6% and 87.9% respectively(p<0.01). However, among patients who received rituximab monoclonal antibody combined with CHOP regimen treatment, it was found that compared with patients with negative PKCδ expression, those with high expression of PKCδ had no statistical difference in either 5-year PFS (72.1% vs 80.0%, p=0.373) or 5-year OS (80.2% vs 83.2%, p=0.127).

**Table 8:** Therapeutic efficacy analysis of the high and low expression groups of PKCδ.

PKCδ	Not achieved CR/PR	Achieved CR/PR	Total	X^2^	p
Low expression	9(6.77)	124(93.23)	133	4.611	0.032^*^
High expression	11(16.42)	56(83.58)	67		
Total	20(10.00)	180(90.00)	200		

*p<0.05, CR, complete response; PR, partial response.

### 3.2 PKCδ gene knockout suppresses tumorigenesis

**Figure 2:**
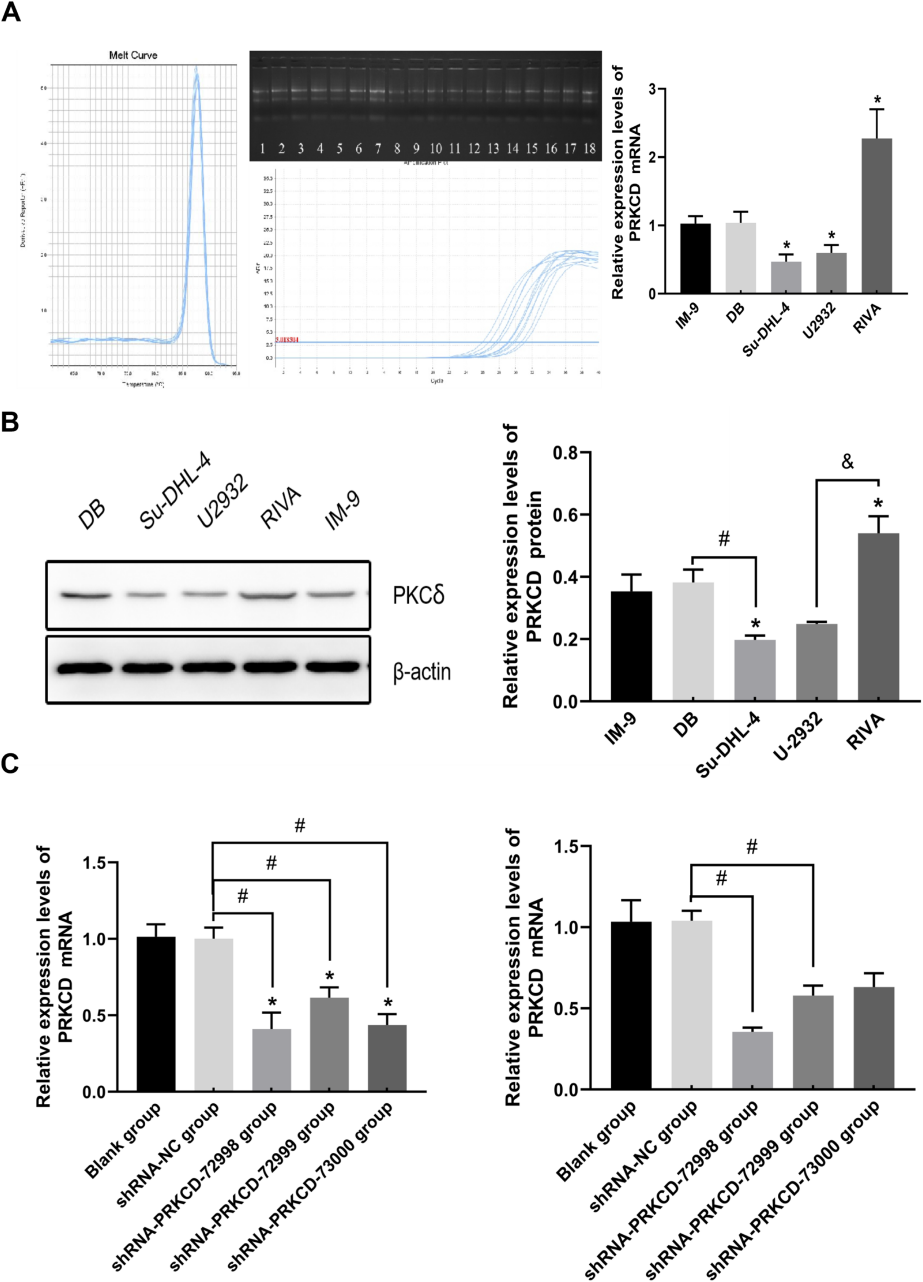
PKCδ is upregulated in DLBCL cell lines and validated after genetic knockdown. **(A)** Relative mRNA expression levels of PRKCD in different DLBCL cell lines (DB, SUDHL-4, U2932, RIVA) and normal IM-9 B lymphocytes was determined by RT-qPCR. Data are normalized to β-actin and presented as mean ± SD of five biological replicates (n=5). Statistical significance was determined by one-way ANOVA with Tukey's *post-hoc* test comparing each cell line to the IM-9 group. *P < 0.05. **(B)** Representative Western blot images (left) and quantitative analysis (right) of PKCδ protein levels in the indicated cell lines. Data are from three independent experiments (n=3) and presented as mean ± SD. Statistical analysis was performed using one-way ANOVA with Tukey's *post-hoc* test. *P < 0.05 compared with the IM-9 group. **(C)** Validation of PRKCD knockdown efficiency by RT-qPCR in DB (left) and RIVA (right) cells transduced with scramble control shRNA (shRNA-NC) or one of three different PRKCD-targeting shRNAs (shRNA-PRKCD-72998, -72999, -73000). Data are presented as mean ± SD of five biological replicates (n=5). Statistical significance was determined by one-way ANOVA with Dunnett's T3 *post-hoc* test comparing each shRNA-PKCδ group to the shRNA-NC group. P < 0.05, ^#^p < 0.05, &p < 0.05.

IM-9 cells and the lymphoma cell lines DB, RIVA, SUDH4 and U2932 entered the logarithmic growth phase on day 3 (**Figure 1C**). The gene expression levels of PKCδ were significantly increased in RIVA and DB cells compared with normal lymphocytes, and significantly decreased in DLBCL cells SUDHL4 and U2932 (**Figures 2A, B**). There are significant differences in the expression levels of the PRKCD gene among different cell lines. Compared with the IM-9 group, there are statistical differences in the SUDHL4, U2932, and RIVA groups, especially the expression levels of the RIVA cell line, which is significantly higher than that of the other cell lines (**Tables 9**, **10**). Original gel files are shown in **Supplementary Figure S1**.

**Table 9:** Baseline expression analysis of the PKCδ gene in five types of cells. (mean ± SD, n=5).

Different groups	PKCδ
IM-9 human peripheral blood B lymphocytes	1.026 ± 0.250
DB-germinal center type	1.038 ± 0.364
Su-DHL-4-germinal center type	0.469 ± 0.241△
U2932-non-germinal center type	0.598 ± 0.258△
RIVA-non-germinal center type	2.273 ± 0.959△

△Compared with the IM-9 group, P<0.05.

**Table 10:** Analysis of PKCδ protein expression levels in various cells (mean ± SD, n=3).

Different groups	PKCδ
IM-9(human peripheral blood B lymphocytes,lymphoblast-like)	0.354 ± 0.092
DB-germinal center type	0.382 ± 0.072
Su-DHL-4-germinal center type	0.198 ± 0.024△
U2932-non-germinal center type	0.249 ± 0.011△
RIVA-non-germinal center type	0.540 ± 0.095△

△Compared with the IM-9 group, P<0.05.

**Figure 3:**
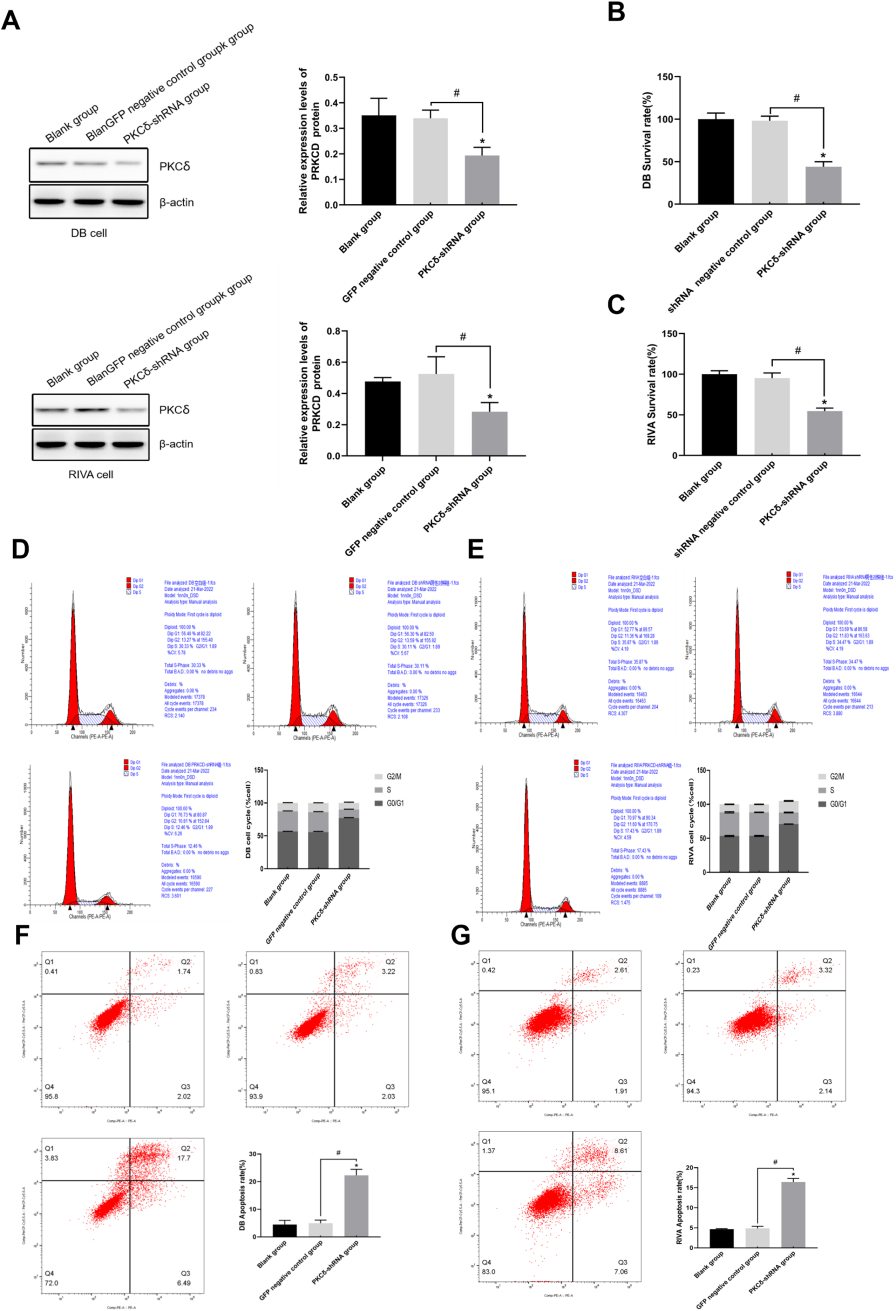
PKCδ knockdown inhibits proliferation, induces cell cycle arrest, and promotes apoptosis in DLBCL cells. **(A)** Western Blot analysis and quantification of PKCδ protein expression in DB (top) and RIVA (bottom) cells from three independent experiments (n=3). Data are presented as mean ± SD. Statistical significance was determined by one-way ANOVA followed by Dunnett's T3 *post-hoc* test; *P<0.05 compared with the Blank group, ^#^P<0.05 compared with the GFP negative control group. **(B, C)** The effect of PKCδ-shRNA on the proliferation of DB cells and RIVA cells. Cell proliferation of DB **(B)** and RIVA **(C)** cells in the Blank, shRNA negative control (shRNA-NC), and PKCδ-shRNA groups was assessed by CCK-8 assay over 5 days. Data points represent the mean ± SD of five biological replicates (n=5). Statistical analysis was performed using two-way ANOVA with Bonferroni's post-test comparing the PKCδ-shRNA group to the other two groups at the 72-hour time point. *P < 0.05. **(D, E)** Cell cycle distribution of DB **(D)** and RIVA **(E)** cells analyzed by flow cytometry with PI staining. The proportion of cells in G0/G1, S, and G2/M phases is shown for the Blank, shRNA-NC, and PKCδ-shRNA groups. Data are from three independent experiments (n=3) and presented as mean ± SD. Significance for the G0/G1 phase population was determined by one-way ANOVA with Tukey's *post-hoc* test. *P < 0.05 compared with the Blank and shRNA-NC groups. **(F, G)** Apoptosis was determined in DB (left) and RIVA cell lines (right) after PKCδ knockdown at different treatment groups by flow cytometry. The bar graph shows the quantification of total apoptotic cells (early + late apoptosis). Data are from three independent experiments (n=3) and presented as mean ± SD. Statistical analysis was performed using one-way ANOVA with Tukey's *post-hoc* test; *P<0.05 compared with the Blank group, P<0.05 compared with the shRNA negative control group.

After infecting cells with viruses carrying three target shRNAs and negative control shRNA. The results showed that compared with the blank group and the negative control group, the three target points of PRKCD knockout could significantly reduce the expression level of PRKCD (**Figure 2C**), especially the shRNA-PRKCD-72998 target point had the most obvious knockout effect(**Table 11**). Subsequent Western Blot and other experiments all utilized this target sequence. Original gel files are shown in **Supplementary Figure S2**. The expression level of PKCδ protein in the blank group was 0.351 ± 0.067. The expression level of PKCδ protein in the GFP negative control group was 0.340 ± 0.032. The expression level of PKCδ protein in the PKCδ-shRNA group was 0.194 ± 0.032. Compared with the blank group and the GFP negative control group, the differences were statistically significant(P<0.05), indicating that PKCδ-shRNA significantly reduced The expression level of PKCδ protein in DB cells (**Figure 3A**, **Table 12**). The expression level of PKCδ protein in the blank group was 0.477 ± 0.025. The expression level of PKCδ protein in the GFP negative control group was 0.525 ± 0.110. The expression level of PKCδ protein in the PKCδ-shRNA group was 0.283 ± 0.059. Compared with the blank group and the GFP negative control group, the differences were statistically significant(P<0.05), indicating that PKCδ-shRNA significantly reduced the expression level of PKCδ protein in RIVA cells (**Table 13**).

**Table 11:** Analysis of PKCδ mRNA expression after shRNA-PKCδ infection in two types of cells (mean ± SD, n=5).

Different groups	DB cell	RIVA cell
Blank group	1.014 ± 0.183	1.034 ± 0.297
shRNA-NC group	1.002 ± 0.161	1.040 ± 0.137
shRNA-PKCδ-72998 group	0.411 ± 0.240△▴	0.355 ± 0.060▴
shRNA-PKCδ-72999 group	0.616 ± 0.152△▴	0.579 ± 0.139▴
shRNA-PKCδ-73000 group	0.437 ± 0.161△▴	0.632 ± 0.189

△Compared with the Blank group, P<0.05; ▴Compared with the shRNA-NC group, P<0.05.

**Table 12:** Analysis of PKCδ protein expression levels in DB cells (mean ± SD, n=3).

Different groups	PKCδ
Blank group	0.351 ± 0.067
GFP negative control group	0.340 ± 0.032
PKCδ-shRNA group	0.194 ± 0.032△▴

^△^Compared with the Blank group, P<0.05; ^▴^Compared with the GFP negative control group, P<0.05.

**Table 13:** Analysis of PKCδ protein expression levels in RIVA cells (mean ± SD, n=3).

Different groups	PKCδ
Blank group	0.477 ± 0.025
GFP negative control group	0.525 ± 0.110
PKCδ-shRNA group	0.283 ± 0.059△▴

△Compared with the Blank group, P<0.05; ▴Compared with the GFP negative control group, P<0.05.

According to the experimental results, 1.5μg/mL puromycin was selected for the screening of stable expression strains in DB and RIVA cells (**Table 14**). To investigate the impact of PRKCD knockout on the proliferation of DB and RIVA cells, the experiment was divided into three groups: the blank group, the shRNA negative control group, and the PKCδ-shRNA group. Each group contained five samples (n=5). The cell viability in the PKCδ-shRNA group was significantly lower than that in the blank group and the shRNA negative control group(P<0.05), indicating that the knockout of PKCδ significantly inhibited the proliferation of DB and RIVA cells and reduced cell viability (**Figures 3B, C**).

**Table 14:** The effect of different concentrations of puromycin on the proliferation of DB and RIVA cells (mean ± SD, n=5).

Puromycin (μg/ml)	Survival rate (%)	
	DB cells	RIVA cells
0	100.000 ± 7.353	100.000 ± 3.054
0.5	35.637 ± 6.797△	56.929 ± 5.928△
1.5	8.420 ± 2.847△▴	7.928 ± 4.449△▴
2.5	7.826 ± 2.740△▴	5.871 ± 2.802△▴

△Compared with puromycin (0 μg/ml), P < 0.05; ▴Compared with puromycin (0.5 μg/ml), P < 0.05.

Cell cycle distribution of DB and RIVA cells under different the blank group, shRNA negative control group, and PKCδ-shRNA group, with each group containing three samples(n=3). The results showed that compared with the blank group and the negative control group, the S phase of the cell cycle was significantly shortened and the G0/G1 phase was prolonged after the knockout of PKCδ, indicating that the cell cycle was arrested after the knockout of PKCδ. PKCδ-shRNA significantly affected the cell cycle distribution of DB cells, leading to a significant increase in the accumulation of cells in the G0/G1 phase and a significant decrease in the distribution in the S phase and G2/M phase (**Figures 3D, E**). This result indicates that PKCδ-shRNA inhibits the proliferation of DB cells by regulating the cell cycle.

In the blank group, the apoptosis rate of DB cells was 4.450 ± 1.527%, and that of RIVA cells was 4.670 ± 0.141%. In the shRNA negative control group, the apoptosis rate of DB cells was 4.960 ± 1.123%, and that of RIVA cells was 4.880 ± 0.510%. Compared with the first two groups, the apoptosis rate in the PKCδ-shRNA group increased significantly, with the apoptosis rate of DB cells being 22.290 ± 2.187% (P<0.05 compared with the blank group); and that of RIVA cells being 16.443 ± 0.879% (P<0.05 compared with the shRNA negative control group). This indicates that the apoptosis rate in the PKCδ-shRNA group was significantly higher than that in the first two groups. The results showed that compared with the blank group and the negative control group, the number of apoptotic cells significantly increased after the knockout of PKCδ, indicating that high expression of PKC promotes the progression of lymphoma (**Figures 3F, G**).

### 3.3 RNA-seq identifies PKCδ-regulated pathways

**Figure 4:**
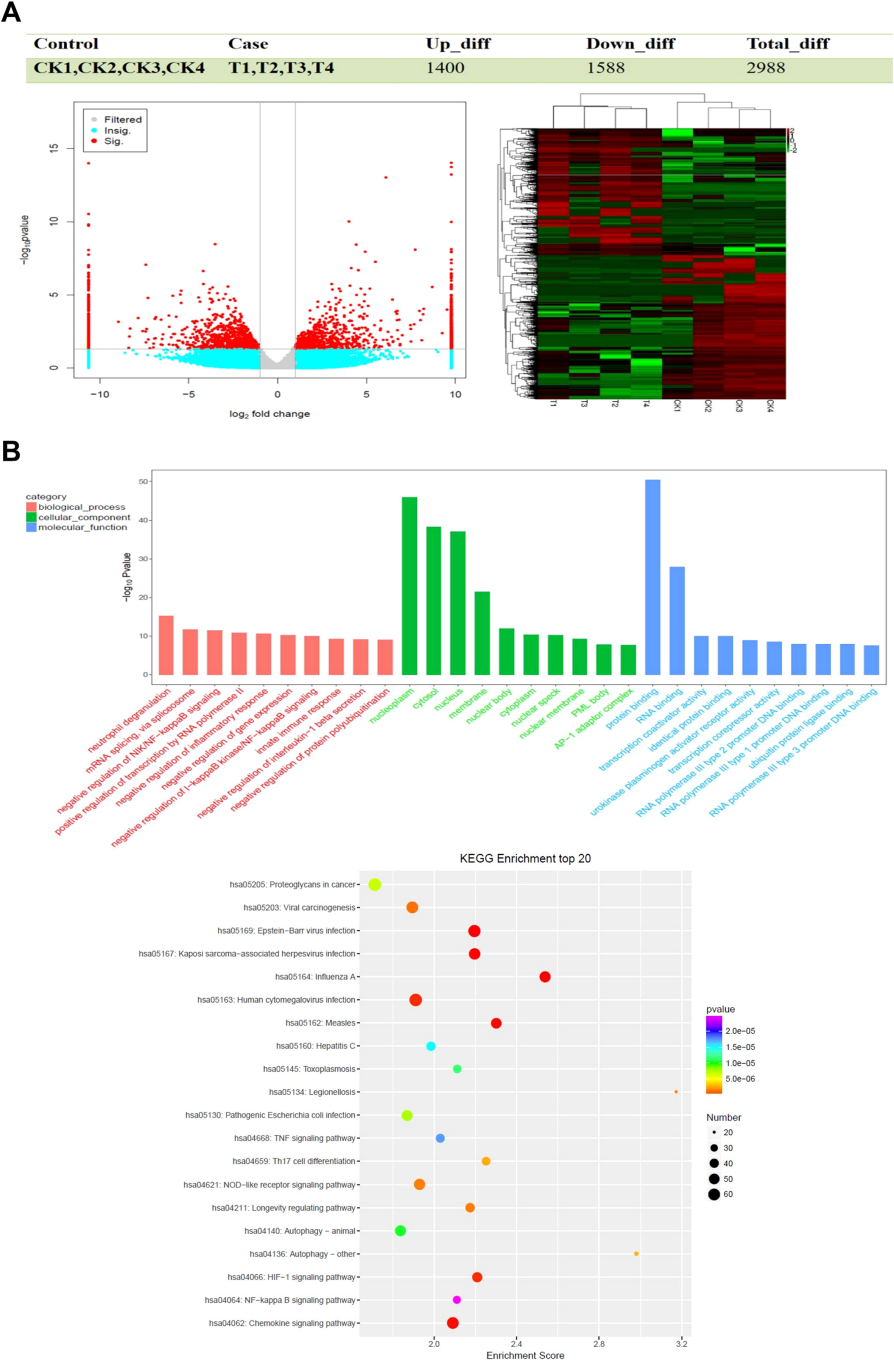
RNA sequencing was used to screen the pathways regulated by PKCδ. **(A)** Left: Volcano plot displaying differentially expressed genes (DEGs) in PRKCD-knockdown DB and RIVA cells (T1-T4) compared to control cells (C1-C4). The thresholds were |log2(Fold Change)| > 1 and adjusted P value < 0.05. Right: Heatmap of hierarchical clustering of the 2,988 DEGs across all samples. **(B)** Gene Ontology (GO) biological process and Kyoto Encyclopedia of Genes and Genomes (KEGG) pathway enrichment analyses of the DEGs. The top significantly enriched terms are shown. The analysis was performed on three to four biological replicates per condition (n=3-4) using the DESeq2 package for DEG identification and the ClusterProfiler R package for enrichment analysis, with a significance threshold of adjusted P value < 0.05.

**Figure 5:**
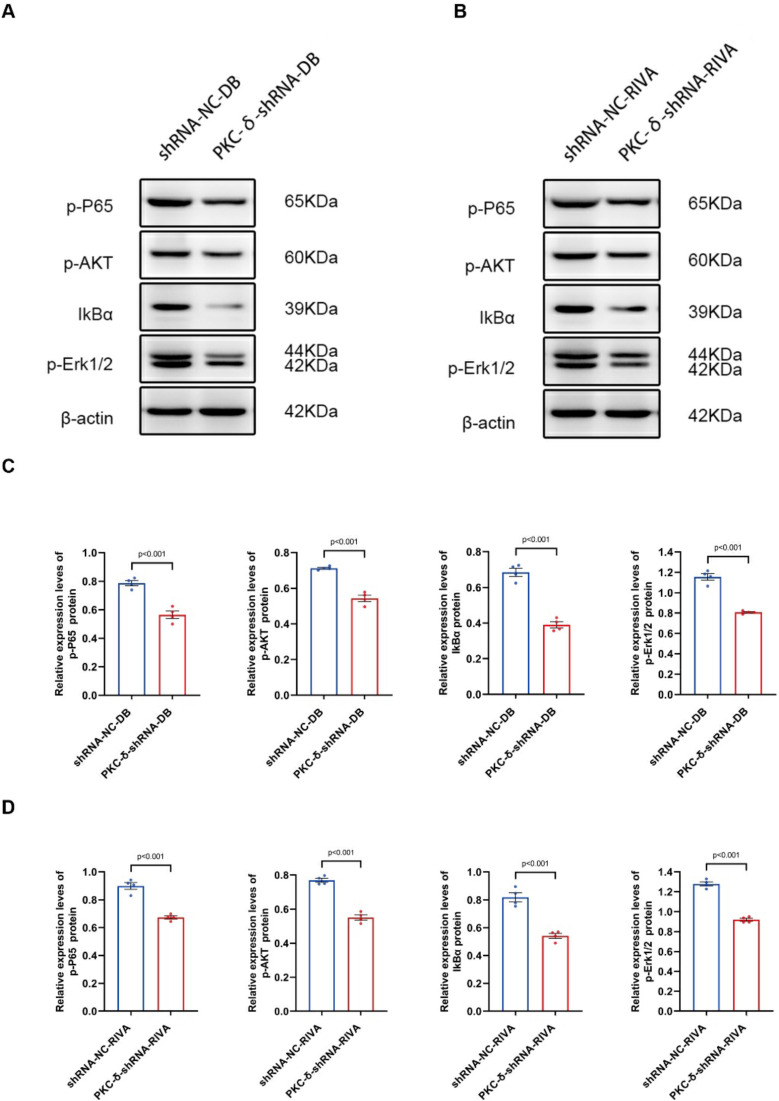
Effects of PKCδ knockdown on the expression of signaling proteins in DB and RIVA cells. Western Blot detection of protein expression bands of p-P65, p-AKT, IkBα, and p-Erk1/2 in DB cells **(A)** and RIVA cells **(B)** after PKCδ knockdown. **(C, D)** Quantitative bar chart analysis of corresponding protein expression levels. Data are presented as mean ± SD of three biological replicates (n=3). shRNA-NC: Negative control lentivirus transfection group; PKCδ-shRNA: PKCδ knockdown lentivirus transfection group.

To elucidate the detailed mechanisms of PKCδ expression in DLBCL drug resistance, DLBCL cell line DB and non-germinal center DLBCL cell line RIVA transfected with PKCδ small interfering RNA lentiviral vectors were used, and candidate differentially expressed genes and signaling pathways were screened using RNA-seq. Volcano plot analysis identified 2,988 differentially expressed genes (DEGs) in PRKCD-knockout DLBCL cells (DB and RIVA) under the threshold of p<0.05 and |log2(Fold Change)|>1, including 1,400 upregulated and 1,588 downregulated genes. Hierarchical clustering of DEGs further revealed a distinct separation between the control group(CK1-CK4) and the PRKCD-knockout group(T1-T4), with coherent expression patterns within each cluster (**Figure 4A**). The volcano plot and clustering analysis showed that downregulated genes were slightly more numerous than upregulated, which may be associated with the suppression of certain signaling pathways. GO analysis indicated that differentially expressed genes were revealed significanly enriched in “centrosome duplication,” “mRNA splicing,” and “AKT/MAPK/NF-κB signaling pathways.” Among them, the significant enrichment of the NF-κB pathway (p<0.05) suggests that PRKCD knockout may enhance chemosensitivity by inhibiting the activity of the IκB kinase (IKK) complex, thereby reducing NF-κB nuclear translocation and subsequently decreasing the expression of pro-survival genes such as Bcl-2 and Survivin. As a core regulatory network for tumor cell survival and drug resistance, the NF-κB pathway may be directly regulated by PKCδ. KEGG analysis further validated pathways associated with cancer drug resistance, including “Proteoglycans in cancer” (hsa05205), “TNF signaling pathway” (hsa04668), and “NF-κB signaling pathway” (hsa04064) (**Figure 4B**). The expression of key signaling proteins in PKCδ knockdown models of DB and RIVA cells was detected. The results showed that in DB cells, compared with the control, PKCδ knockdown significantly decreased the protein expression levels of p-P65 (0.786 ± 0.036 vs. 0.565 ± 0.053, P<0.001), p-AKT (0.712 ± 0.010 vs. 0.543 ± 0.036, P<0.001), IkBα (0.684 ± 0.046 vs. 0.390 ± 0.034, P<0.001), and p-Erk1/2 (1.155 ± 0.065 vs. 0.807 ± 0.015, P<0.001) (**Figures 5A, C**). Similarly, in RIVA cells, PKCδ knockdown significantly inhibited the expression of p-P65 (0.899 ± 0.049 vs. 0.673 ± 0.023, P<0.001), p-AKT (0.769 ± 0.024 vs. 0.551 ± 0.031, P<0.001), IkBα (0.818 ± 0.064 vs. 0.541 ± 0.036, P<0.001), and p-Erk1/2 (1.280 ± 0.043 vs. 0.919 ± 0.030, P<0.001) (**Figures 5B, D**). Original gel files are shown in **Supplementary Figure S3**. These results consistently indicate that downregulation of PKCδ can significantly inhibit the activity of the NF-κB, AKT, and MAPK signaling pathways.

### 3.4 PKCδ knockout enhances drug sensitivity/chemosensitivity

**Figure 6:**
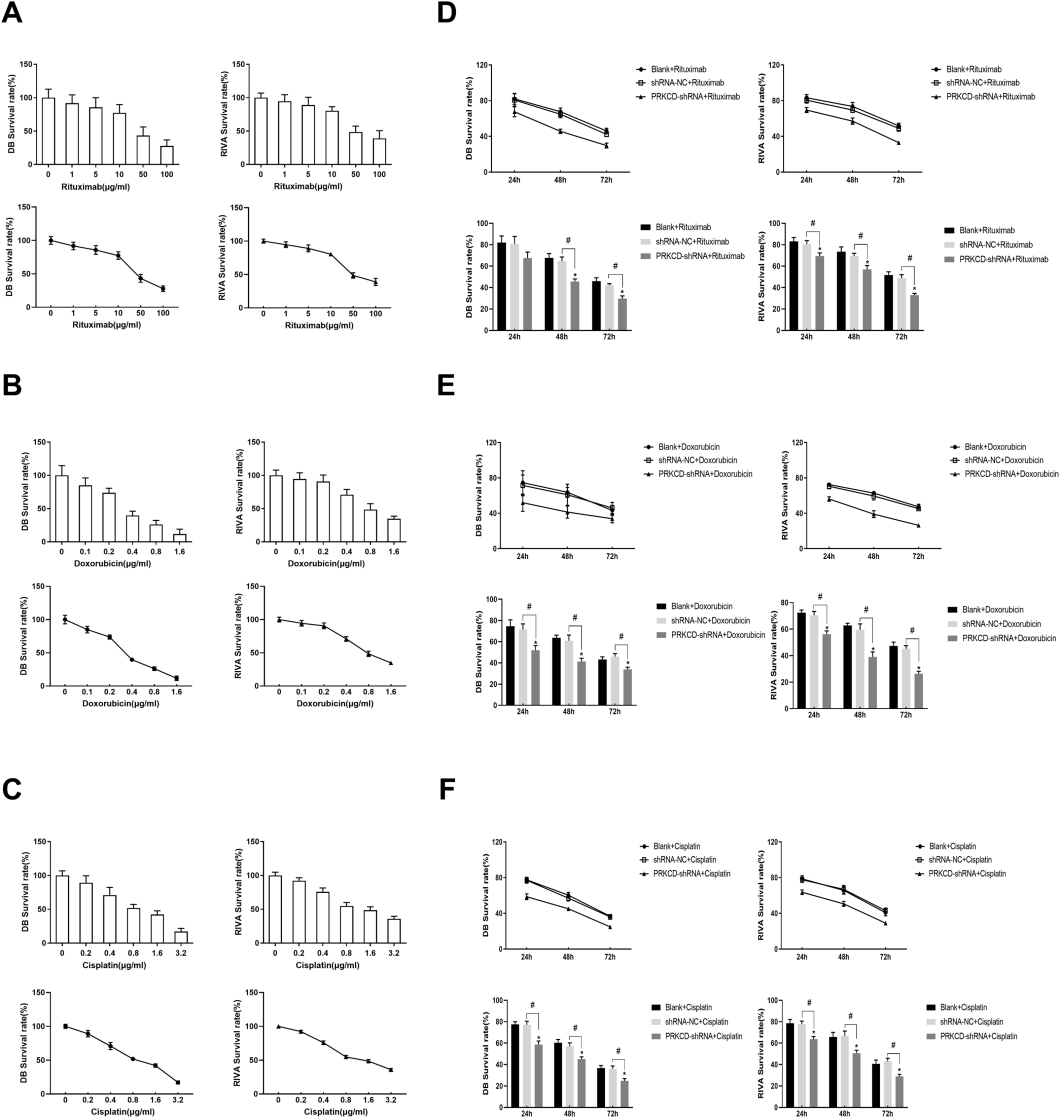
PKCδ knockout enhanced the sensitivity of DLBCL cells to chemotherapeutic drugs. **(A–C)** Dose-response curves showing the survival rate of DB and RIVA cells treated with increasing concentrations of **(A)** rituximab, **(B)** doxorubicin, and **(C)** cisplatin for 72 hours, as determined by CCK-8 assay. Data points represent the mean ± SD of at least three independent experiments, each with technical triplicates (n≥3). The half-maximal inhibitory concentration (IC50) for each drug was calculated using non-linear regression. **(D–F)** Bar charts showing the cell survival rates of DB and RIVA cells in Blank, shRNA-NC, and PRKCD-shRNA groups after treatment with non-toxic IC25 doses of **(D)** rituximab, **(E)** doxorubicin, or **(F)** cisplatin for 24, 48, and 72 hours. Data are presented as mean ± SD of three biological replicates (n=3). Statistical significance was determined by two-way ANOVA with Bonferroni’s post-test to compare the PRKCD-shRNA group to the shRNA-NC group at each time point. ^#^P < 0.05.

The IC50 values of doxorubicin, cisplatin, and rituximab for DB cells determined by the CCK8 method were 0.356μg/ml, 0.964μg/ml, and 35.945μg/ml, respectively. The IC50 values of doxorubicin, cisplatin, and rituximab for RIVA cells were 0.871μg/ml, 1.444μg/ml, and 53.601μg/ml, respectively. We first co-cultured PRKCD-knockout cell lines with different concentrations of drugs(rituximab, doxorubicin, cisplatin)and assessed drug efficacy by cell viability. The data indicated that as the drug concentration increased, the viability of both DB and RIVA cells significantly decreased(p<0.05), and DB cells generally exhibited higher drug sensitivity than RIVA cells. Dose-dependent decreases in cell viability were observed for all tested drugs, with DB cells showing higher sensitivity, particularly to doxorubicin and cisplatin (**Figures 6A–C**).

We further explored the time-dependent effects of drug efficacy and validated whether PRKCD knockout enhances the therapeutic effects of doxorubicin, cisplatin, and rituximab by comparing the differences in cell proliferation inhibition among different treatment groups (blank control, shRNA-NC, PRKCD-shRNA)to clarify the regulatory role of PKCδ. The combination of rituximab and PRKCD knockout led to a gradual decrease in DB cell viability from 80% at 24h to 40% at 72h, while the control groups maintained higher viability (**Figure 6D**). Similar trends were observed for with doxorubicin and cisplatin (**Figures 6E, F**), indicating that prolonged exposure amplifies the therapeutic efficacy of targeting PKCδ. PRKCD knockout significantly reduced cell proliferation rates in both DB and RIVA cells when combined with doxorubicin, cisplatin, or rituximab (**Figures 6D–F**). The inhibitory effects were most pronounced at 72h, with the PRKCD-shRNA groups showing the lowest survival rates. These results demonstrate that the depletion of PKCδ synergizes with chemotherapeutic drugs in a time-dependent manner.

### 3.5 *In Vivo* validation

**Figure 7:**
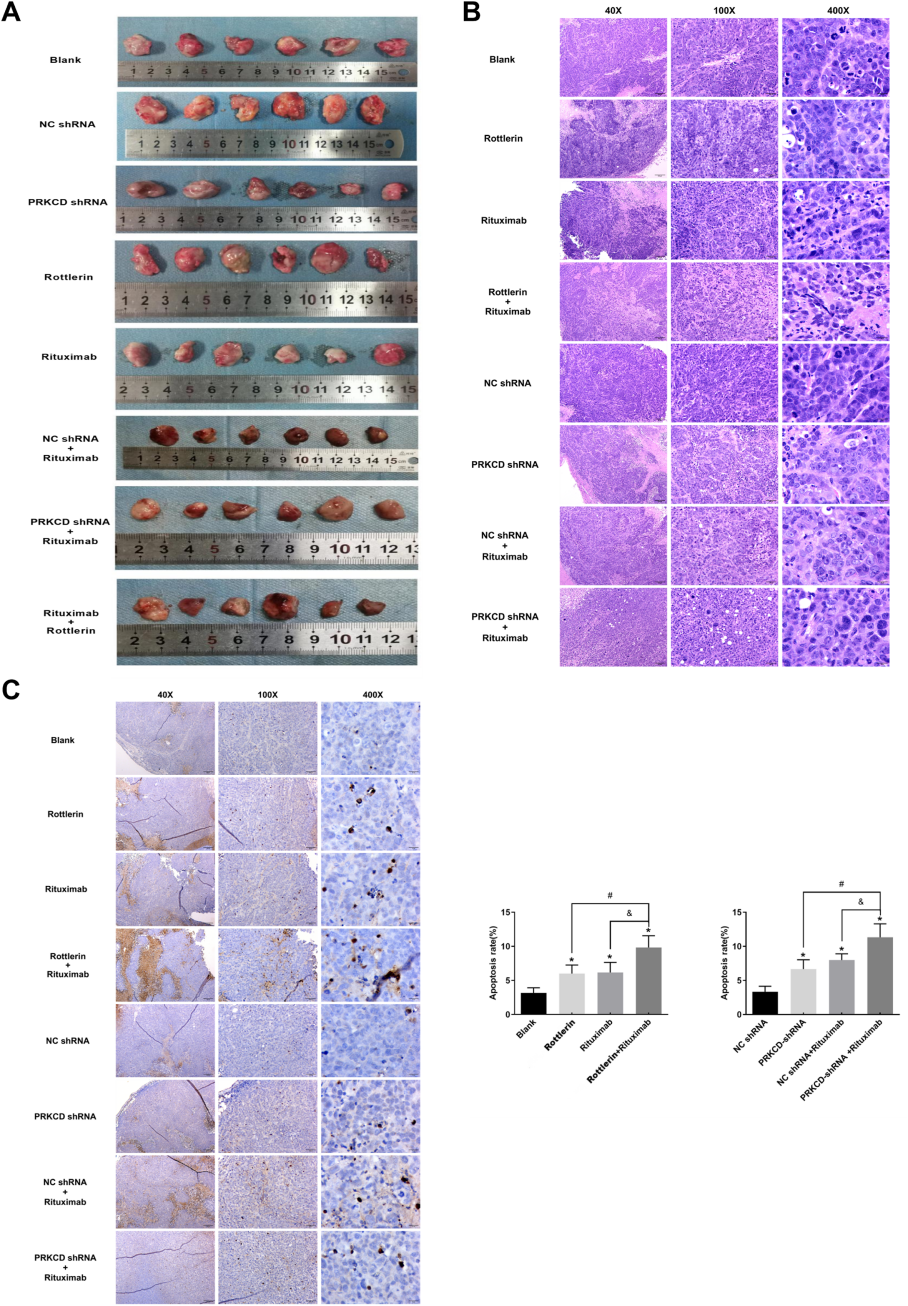
Rottlerin inhibits tumor growth and enhances the efficacy of rituximab *in vivo*. **(A)** Measurement and changes of tumor volume in female nude mice across different treatment groups. **(B)** Representative hematoxylin and eosin (H&E) staining of tumor tissues from the indicated groups. Scale bar, 50 μm. Images are representative of tumors from five female nude mice per group (n=5). **(C)** Representative TUNEL staining (brown nuclei indicate apoptotic cells) of tumor tissues from the indicated groups. Scale bar, 50 μm. The bar graph (right) shows the quantitative analysis of the TUNEL-positive cell rate. Data are presented as mean ± SD from five independent tumor samples per group (n=5). Statistical significance was determined by one-way ANOVA with Tukey’s *post-hoc* test. *P < 0.05 compared with the Blank control group. ^#^p < 0.05, &p < 0.05.

We assessed the efficacy of PKCδ small interfering RNA (shRNA) transfection, rottlerin, rituximab, and their combinations. Compared with the control group, all treatment groups receiving PKCδ intervention (including shRNA transfection and Rottlerin treatment) exhibited a pronounced inhibitory effect on tumor growth. Specifically, the growth rate of subcutaneous xenografts in these treatment groups was significantly curtailed, and the tumor volume was markedly smaller than that in the control group (**Figure 7A**). Among them, the combined treatment group with PKCδ shRNA lentiviral vector transfection, Rottlerin, rituximab, and chemotherapy demonstrated the most remarkable tumor-inhibiting effect, achieving an inhibition rate of 65.85% (**Table 15**), which was significantly superior to that of other groups (P<0.001). Tumor growth was the most sluggish in this group, and some nude mice even exhibited tumor regression. Notably, the combination of Rottlerin and rituximab yielded an effect analogous to that of the PRKCD-shRNA group, thereby providing supportive evidence for the therapeutic potential of targeting PKCδ. Tumor tissues from the PRKCD knockout group displayed extensive eosinophilic red-stained areas, indicative of tumor cell death. The cells were loosely arranged, with reduced tumor cell density and diminished atypia. A decrease in mitotic figures suggested suppressed proliferative activity. In stark contrast, tumors from the control group exhibited densely packed, highly atypical cells with active mitosis and minimal necrotic areas (**Figure 7B**). TUNEL staining revealed a marked increase in positive cells (dense brown nuclear staining) in the treatment groups, indicating a significantly higher apoptosis rate compared with the control group. Compared with the blank control group, all treatment groups demonstrated increased apoptosis to varying degrees in the tumor tissues (**Figure 7C**). The apoptosis rate was the highest in the group treated with the combination of rituximab and Rottlerin, reaching 9.83%, which was significantly higher than that of other groups (P<0.05). Only sporadic apoptotic cells were observed in the control group, suggesting that drug-resistant tumor cells had evaded apoptosis.

**Table 15:** Comparison of tumor growth inhibition rate (IR) among different groups.

Experimental groups	Tumor growth inhibition rate (%) (IR)
Rottlerin treatment group	39.14
Rituximab treatment group	39.26
Rottlerin + Rituximab group	65.85
Negative control shRNA group	7.43
NC shRNA + Rituximab group	39.49
PRKCD-shRNA group	35.89
PRKCD-shRNA + Rituximab group	64.92

## 4 Discussion

It has been confirmed that in adenoid cystic carcinoma, renal cell carcinoma, and gastrointestinal stromal tumors, the expression of PKCδ is elevated, and elevated PKCδ expression is an adverse prognostic factor for tumors (20). This is consistent with the experimental results of this study. This study demonstrates that high expression of PKCδ in DLBCL is associated with poor overall and progression-free survival in patients, PKCδ overexpression is an independent adverse prognostic factor in DLBCL, correlating with aggressive disease and chemoresistance. Our patient cohort includes individuals who received only CHOP treatment without rituximab, primarily due to economic reasons. While our cohort reflects the inherent biases of clinical practice, the balance in most baseline features and, crucially, the robust multivariate analysis demonstrate that the prognostic role of PKCδ and its interaction with rituximab are genuine effects. The Reyland laboratory has confirmed that PKCδ is a synthetic lethal target for certain K-Ras mutant cancers (21). In certain non-small cell lung cancer (NSCLC) cells, the maintenance of ERK activation downstream of mutant K-Ras requires PKCδ, which may underlie resistance to tyrosine kinase inhibitors (22, 23). In lymphoma, PKCδ activation stimulates anticancer drug-mediated apoptosis through caspase-3 activation (24, 25), JNK activation (26), or phosphorylation and activation of lysosomal acidic sphingomyelinase. We have investigated the chemical inhibition of PKCδ in DLBCL cell lines, After knocking out PKCδ, the tested DLBCL cell lines showed a significant increase in apoptosis *in vitro*, exhibiting a time- and dose-dependent response. The study found that blocking PKCδ has been shown to improve intra-tumor T cell infiltration and inhibit tumor expansion (27). Mechanistically, PKCδ promotes tumorigenesis via AKT/MAPK/NF-κB pathway activation, consistent with its role in solid tumors (28). After PKCδ knockout, the cell cycle S phase was significantly shortened, and the G0/G1 phase was prolonged, indicating that PKCδ can drive cell cycle arrest in the G1 phase, which is consistent with previous findings (29). A block in the S phase has been observed as a consequence of PKCδ overexpression. Several findings underline the PKCδ involvement in apoptosis as a response to chemotherapy (30).

By integrating differential expression analysis of RNA-seq data, functional enrichment, and pathway analysis, this study systematically elucidated the potential molecular mechanisms of PRKCD knockout in DLBCL drug resistance. The study identified some extremely differentially expressed genes and virus-related pathways(such asEpstein-Barr virus infection, hsa05169). The enrichment of the NF-κB and TNF signaling pathways can participate in tumorigenesis and metastasis, tumor cell survival, immune evasion, or microenvironment regulation, especially carcinogenic mechanisms related to the NF-κB pathway. It has been reported that the interaction between PKCδ and CARMA1 impairs NF-κB activation (31). The NF-κB pathway is a core regulatory network for inflammation responses and cell survival, and its enrichment suggests that PKCδ may regulate inflammatory factors, anti-apoptotic signals, or the immune microenvironment through this pathway, thereby affecting the development of drug resistance in DLBCL. For example, targeting the PKCδ-NF-κB axis or combining with AKT inhibitors may reverse the drug-resistant phenotype (32–34).

Although some studies claim to have identified specific inhibitors of PKCδ, there are still many challenges in practical application. One major issue is off-target effects (35), that is, the inhibitors may also act on other PKC isoforms with structures similar to PKCδ. Some of these isoforms may have physiological functions opposite to those of PKCδ, thereby weakening or even canceling the expected effects of inhibiting PKCδ. Therefore, exploring the potential of acute PKCδ inhibition via specific pharmacological agents to achieve comprehensive tumor progression control presents a compelling avenue for future research. Our results substantiate clinical exploration of this combinatorial approach. Knockout of PKCδ effectively enhances *in vivo* sensitivity of germinal center–type DLBCL cells to rituximab and chemotherapeutic agents, resulting in significant inhibition of tumor growth. Our finding indicates that PKCδ activity is closely associated with drug resistance in DLBCL. *In vivo* experimental results strongly support our central hypothesis: PKCδ is a key factor mediating resistance in germinal center–type DLBCL.

The concordant *in vitro* and *in vivo* results suggest that PKCδ promotes DLBCL progression by enhancing cell survival and drug resistance. The efficacy of both genetic knockdown (PKCδ-shRNA) and pharmacological inhibition with Rottlerin in suppressing tumor growth and synergizing with rituximab underscores PKCδ as a viable therapeutic target. However, the genetic evidence provides the most compelling support for PKCδ’s specific role. Subsequent studies need to detect intracellular PKCδ protein levels through functional experiments (such as ChIP-qPCR, gene knockout/overexpression) to verify the direct regulatory effect of PKCδ on pathways such as NF-κB from the direction of gene expression changes, and analyze the activation or inhibition status of the pathways. If PKCδ is not directly enriched, its potential association with differentially expressed genes needs to be analyzed through protein interaction networks. The efficacy of targeted interventions should be assessed using organoids or mouse models to promote translational applications, potentially providing new biomarkers for DLBCL drug resistance. There are differences in the regulatory networks of PKCδ among different DLBCL subtypes (such as ABC and GCB). In the future, multi-omics analysis (genomics, transcriptomics, and phosphoproteomics) should be integrated to screen for the optimal patient population that will benefit the most (36).

While considering the translational potential of our findings, it’s important to recognize several limitations. The pharmacological validation of PKCδ’s role relied on Rottlerin, a compound known for its off-target effects on other kinases. Although the concordance between Rottlerin treatment and our specific PKCδ-shRNA knockdown data strengthens our conclusions, future studies should utilize more specific PKCδ inhibitors or alternative strategies to fully confirm these pharmacological effects. The absence of sufficient clinical data prevents us from comparing survival or immunotherapeutic efficacy in DLBCL patients treated with or without Rottlerin. The study of the mechanisms involved in the pro-apoptotic responses mediated by PKCδ to different chemotherapeutic drugs could be of great impact in the design of PKCδ-specific inhibitors for cancer treatment.

## Data Availability

The original contributions presented in the study are included in the article/**Supplementary Material**. Further inquiries can be directed to the corresponding authors.
