# Supplementary material for: Research on the correlation and potential mechanism of PKCδ expression with efficacy and prognosis in diffuse large B-cell lymphoma
Source: Front Oncol. 2026 Feb 6;16:1690426. doi: 10.3389/fonc.2026.1690426 (PMC12921706; doi:10.3389/fonc.2026.1690426)
Supplement: Supplementary file 1 [file DataSheet1.docx]

Supplementary Material

#
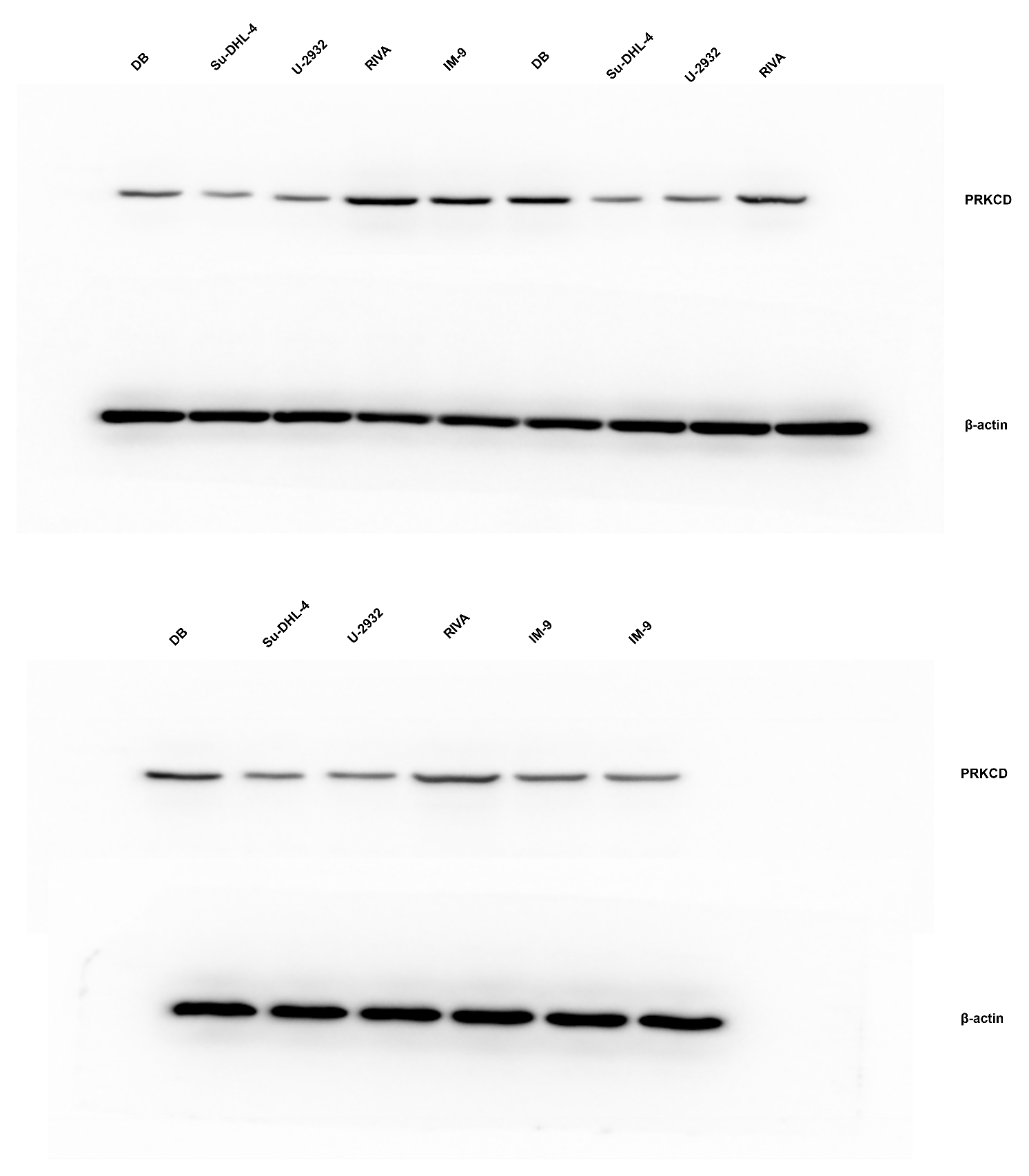


**Supplementary Figure 1.** Expression levels of PKCδ protein among different cell lines(DB, Su-DHL-4, U-2932, RIVA, IM-9). The experiment was repeated three times.

**
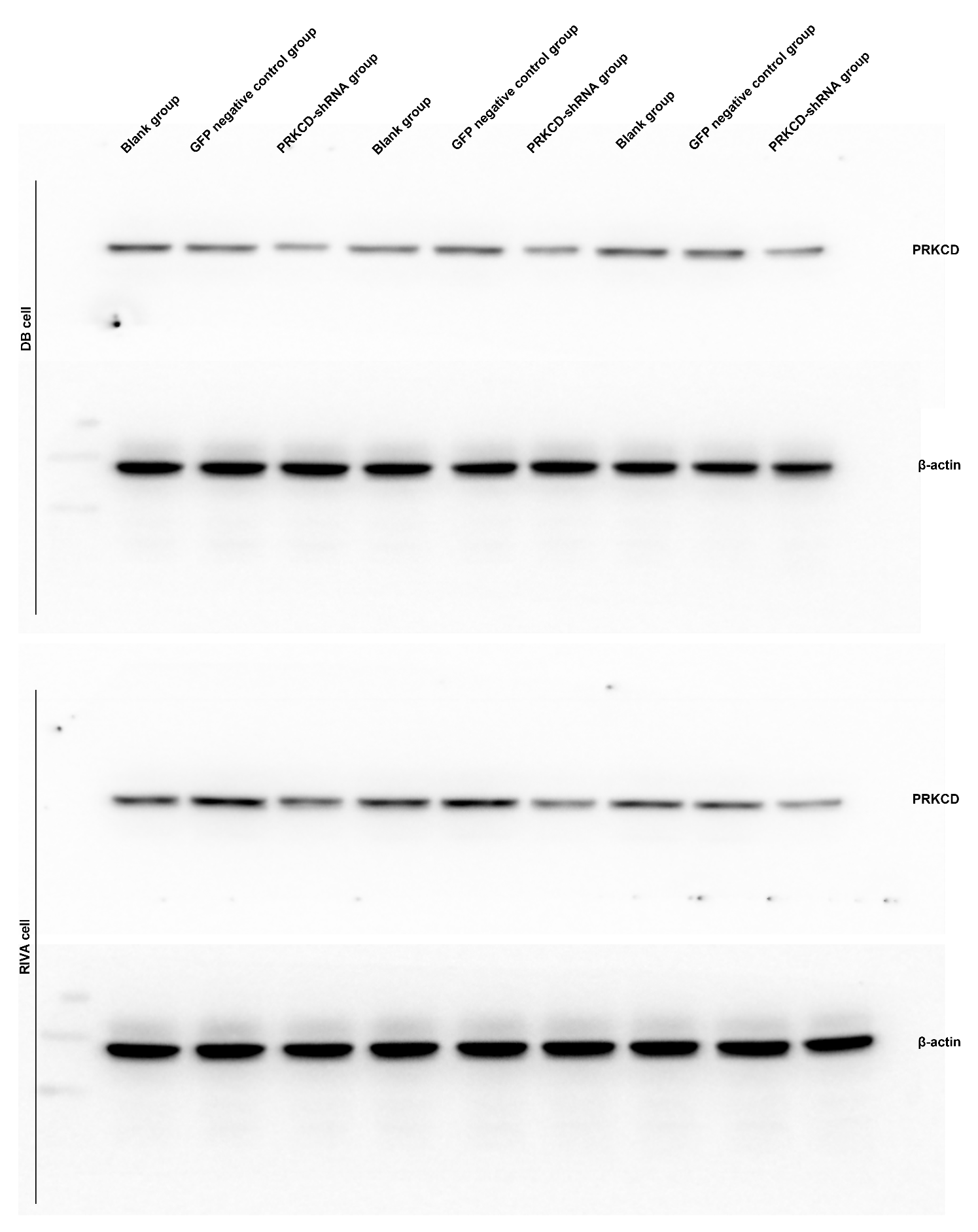
**

**Supplementary Figure 2.** The expression levels of PKCδ in the blank control group, the PKCδ-knockout group, and the negative control group. The experiment was repeated three times.


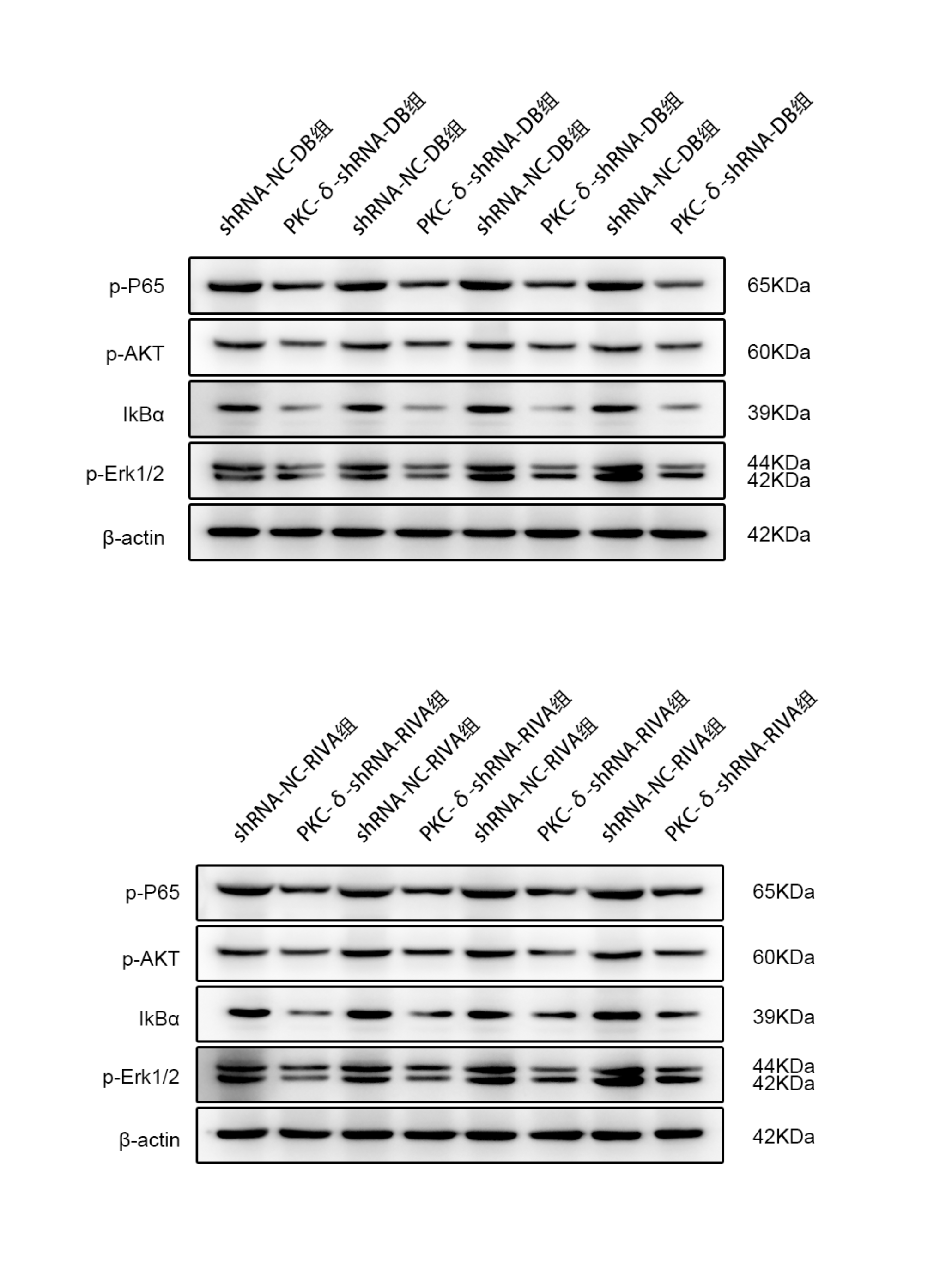


**Supplementary Figure 3.** Western Blot detection of protein expression bands of p-P65, p-AKT, IkBα, and p-Erk1/2 in DB cells (up) and RIVA cells (down) after PKCδ knockdown. The experiment was repeated three times.

**Supplementary table S1** Comparison of Baseline Characteristics Between CHOP and R-CHOP group.

|  | CHOP group | R-CHOP group | χ² | p |
| --- | --- | --- | --- | --- |
| sex |  |  | 0.05 | 0.831 |
| female | 43 (39.8%) | 65 (60.2%) |  |  |
| male | 38 (41.3%) | 54 (58.7%) |  |  |
| Age |  |  | 0.12 | 0.728 |
| ≤60 | 61 (41.2%) | 87 (58.8%) |  |  |
| >60 | 20 (38.5%) | 32 (61.5%) |  |  |
| Stage |  |  | 2.93 | 0.087 |
| I-II | 44 (46.8%) | 50 (53.2%) |  |  |
| Ⅲ-Ⅳ | 37 (34.9%) | 69 (65.1%) |  |  |
| IPI |  |  | 8.40 | 0.038 |
| 0-1 | 50 (50.5%) | 49 (49.5%) |  |  |
| 2 | 15 (28.3%) | 38 (71.7%) |  |  |
| 3 | 13 (33.3%) | 26 (66.7%) |  |  |
| 4-5 | 3 (33.3) | 6 (66.7%) |  |  |
| Extranodal involvement |  |  | 5.71 | 0.017 |
| 0-1 | 69 (45.1%) | 84 (54.9%) |  |  |
| ≥2 | 12 (25.5%) | 35 (74.5%) |  |  |
| cycles |  |  | 2.51 | 0.286 |
| ＜4 | 8 (29.6%) | 19 (70.4%) |  |  |
| 4-7 | 60 (44.1%) | 76 (55.9%) |  |  |
| ≥8 | 13 (35.1%) | 24 (64.9%) |  |  |
| LDH level |  |  | 0.68 | 0.409 |
| normal | 53 (42.7%) | 71 (57.3%) |  |  |
| abnormal | 28 (36.8%) | 48 (63.2%) |  |  |
| ECOG score |  |  | 6.21 | 0.013 |
| 0-1 | 11 (24.4%) | 34 (75.6%) |  |  |
| ≥2 | 70 (45.2%) | 85 (54.8%) |  |  |
| PKCδ expression |  |  | 0.07 | 0.792 |
| negtive | 53 (39.8%) | 80 (60.2%) |  |  |
| positive | 28 (41.8%) | 39 (58.2%) |  |  |
| Histologic category |  |  | 0.04 | 0.837 |
| GCB | 40 (41.2%) | 57 (58.8%) |  |  |
| Non-GCB | 41 (39.8%) | 62 (60.2%) |  |  |
